# On the Stability of One-Dimensional Wave Equation

**DOI:** 10.1155/2013/978754

**Published:** 2013-10-27

**Authors:** Soon-Mo Jung

**Affiliations:** Mathematics Section, College of Science and Technology, Hongik University, Sejong 339-701, Republic of Korea

## Abstract

We prove the generalized Hyers-Ulam stability of the one-dimensional wave equation, *u*_*tt*_ = *c* ^2^ *u*_*xx*_, in a class of twice continuously differentiable functions.

## 1. Introduction

In 1940, Ulam [1] gave a wide ranging talk before the mathematics club of the University of Wisconsin in which he discussed a number of important unsolved problems. Among those was the question concerning the stability of group homomorphisms:

* Let *G*_1_ be a group and let *G*_2_ be a metric group with the metric *d*(·, ·). Given *ε* > 0, does there exist a *δ* > 0 such that if a function *h* : *G*_1_ → *G*_2_ satisfies the inequality *d*(*h*(*xy*), *h*(*x*)*h*(*y*)) < *δ*, for all *x*, *y* ∈ *G*_1_, then there exists a homomorphism *H* : *G*_1_ → *G*_2_ with *d*(*h*(*x*), *H*(*x*)) < *ε*, for all *x* ∈ *G*_1_? *

The case of approximately additive functions was solved by Hyers [2] under the assumption that *G*_1_ and *G*_2_ are Banach spaces. Indeed, he proved that each solution of the inequality ||*f*(*x* + *y*) − *f*(*x*) − *f*(*y*)|| ≤ *ε*, for all *x* and *y*, can be approximated by an exact solution, say an additive function. In this case, the Cauchy additive functional equation, *f*(*x* + *y*) = *f*(*x*) + *f*(*y*), is said to have the Hyers-Ulam stability.

Rassias [3] attempted to weaken the condition for the bound of the norm of the Cauchy difference as follows:

(1) $$\begin{array}{c} ||f\left(x+y\right)-f\left(x\right)-f\left(y\right)||\leq \varepsilon \left({||x||}^{p}+{||y||}^{p}\right) \end{array}$$

and proved Hyers' theorem. That is, Rassias proved the generalized Hyers-Ulam stability (or Hyers-Ulam-Rassias stability) of the Cauchy additive functional equation. Since then, the stability of several functional equations has been extensively investigated [4–9].

The terminologies, the generalized Hyers-Ulam stability, and the Hyers-Ulam stability can also be applied to the case of other functional equations, differential equations, and various integral equations. 

Given a real number *c* > 0, the partial differential equation

(2) $$\begin{array}{c} u_{tt}\left(x,t\right)-c^{2}u_{xx}\left(x,t\right)=0 \end{array}$$

is called the (one-dimensional) wave equation, where *u*_*tt*_(*x*, *t*) and *u*_*xx*_(*x*, *t*) denote the second time derivative and the second space derivative of *u*(*x*, *t*), respectively.

Let *φ* : ℝ × ℝ → [0, *∞*) be a function. If, for each twice continuously differentiable function *u* : ℝ × ℝ → *ℂ* satisfying

(3) $$\begin{array}{c} \left|u_{tt}\left(x,t\right)-c^{2}u_{xx}\left(x,t\right)\right|\leq \varphi \left(x,t\right)\;\left(x,t\in \mathbb{R}\right), \end{array}$$

there exist a solution *u*_0_ : ℝ × ℝ → *ℂ* of the (one-dimensional) wave equation (2) and a function Φ : ℝ × ℝ → [0, *∞*) such that

(4) $$\begin{array}{c} \left|u\left(x,t\right)-u_{0}\left(x,t\right)\right|\leq \Phi \left(x,t\right)\;\left(x,t\in \mathbb{R}\right), \end{array}$$

where Φ(*x*, *t*) is independent of *u*(*x*, *t*) and *u*_0_(*x*, *t*), then we say that the wave equation (2) has the generalized Hyers-Ulam stability (or the Hyers-Ulam-Rassias stability).

In this paper, using an idea from [10], we prove the generalized Hyers-Ulam stability of the (one-dimensional) wave equation (2).

## 2. Generalized Hyers-Ulam Stability

In the following theorem, using the d'Alembert method (method of characteristic coordinates), we prove the generalized Hyers-Ulam stability of the (one-dimensional) wave equation (2).

**Theorem 1**

Let a function *φ* : ℝ × ℝ → [0, *∞*) be given such that the double integral

(5) $$\begin{array}{c} \overset{b}{\underset{0}{\int }}\overset{a}{\underset{0}{\int }}\varphi \left(\frac{\mu +\nu }{2},\frac{\mu -\nu }{2c}\right)d\mu d\nu \end{array}$$

exists for all *a*, *b* ∈ ℝ. If a twice continuously differentiable function *u* : ℝ × ℝ → *ℂ* satisfies the inequality

(6) $$\begin{array}{c} \left|u_{tt}\left(x,t\right)-c^{2}u_{xx}\left(x,t\right)\right|\leq \varphi \left(x,t\right) \end{array}$$

for all *x*, *t* ∈ ℝ, then there exists a solution *u*_0_ : ℝ × ℝ → *ℂ* of the wave equation (2) which satisfies

(7) |u(x,t)−u0(x,t)| ≤14c2|∫0x−ct∫0x+ctφ(μ+ν2,μ−ν2c)dμdν|

for all *x*, *t* ∈ ℝ.

**Proof**

Let us define a function *v* : ℝ × ℝ → *ℂ* by

(8) $$\begin{array}{c} v\left(w,z\right):=u\left(\frac{w+z}{2},\frac{w-z}{2c}\right). \end{array}$$

If we set *w* = *x* + *ct* and *z* = *x* − *ct*, then we have *u*(*x*, *t*) = *v*(*w*, *z*) and

(9) ut(x,t)=vw(w,z)∂w∂t+vz(w,z)∂z∂t=cvw(w,z)−cvz(w,z),utt(x,t)  =cvww(w,z)∂w∂t+cvwz(w,z)∂z∂t −cvzw(w,z)∂w∂t−cvzz(w,z)∂z∂t=c2vww(w,z)−2c2vwz(w,z)+c2vzz(w,z),ux(x,t)=vw(w,z)∂w∂x+vz(w,z)∂z∂x=vw(w,z)+vz(w,z),uxx(x,t)=vww(w,z)∂w∂x+vwz(w,z)∂z∂x +vzw(w,z)∂w∂x+vzz(w,z)∂z∂x=vww(w,z)+2vwz(w,z)+vzz(w,z),

for all *x*, *t* ∈ ℝ. Hence, we have

(10) $$\begin{array}{c} u_{tt}\left(x,t\right)-c^{2}u_{xx}\left(x,t\right)=-4c^{2}v_{wz}\left(w,z\right), \end{array}$$

for any *x*, *t* ∈ ℝ. Thus, it follows from inequality (6) that

(11) $$\begin{array}{c} \left|v_{wz}\left(w,z\right)\right|\leq \frac{1}{4c^{2}}\varphi \left(\frac{w+z}{2},\frac{w-z}{2c}\right), \end{array}$$

for any *w*, *z* ∈ ℝ.

Therefore, we get

(12) −14c2∫0z∫0wφ(μ+ν2,μ−ν2c)dμdν  ≤∫0z∫0wvwz(μ,ν)dμdν  ≤14c2∫0z∫0wφ(μ+ν2,μ−ν2c)dμdν

or equivalently

(13) |v(w,z)−v(w,0)−v(0,z)+v(0,0)| ≤14c2|∫0z∫0wφ(μ+ν2,μ−ν2c)dμdν|,

for all *w*, *z* ∈ ℝ.

On account of (8), we get

(14) $$\begin{array}{c} v\left(w,z\right)=u\left(\frac{w+z}{2},\frac{w-z}{2c}\right),\;\;v\left(w,0\right)=u\left(\frac{w}{2},\frac{w}{2c}\right),\\ v\left(0,z\right)=u\left(\frac{z}{2},-\frac{z}{2c}\right),\;\;v\left(0,0\right)=u\left(0,0\right). \end{array}$$

Hence, it follows from (13) and the last equalities that

(15) |u(w+z2,w−z2c)−u(w2,w2c)−u(z2,−z2c)+u(0,0)| ≤14c2|∫0z∫0wφ(μ+ν2,μ−ν2c)dμdν|,

for all *w*, *z* ∈ ℝ.

If we set *w* = *x* + *ct* and *z* = *x* − *ct* in the last inequality, then we obtain

(16) |u(x,t)−u0(x,t)| ≤14c2|∫0x−ct∫0x+ctφ(μ+ν2,μ−ν2c)dμdν|,

for all *x*, *t* ∈ ℝ, where we set

(17) u0(x,t):=u(x2+c2t,x2c+t2)  +u(x2−c2t,−x2c+t2)−u(0,0).

By some tedious calculations, we get

(18) ∂∂tu0(x,t) =c2ux(x2+ct2,x2c+t2)+12ut(x2+ct2,x2c+t2)   −c2ux(x2−ct2,−x2c+t2)+12ut(x2−ct2,−x2c+t2),∂2∂t2u0(x,t) =c24uxx(x2+ct2,x2c+t2)+c2uxt(x2+ct2,x2c+t2)   +14utt(x2+ct2,x2c+t2)+c24uxx(x2−ct2,−x2c+t2)   −c2uxt(x2−ct2,−x2c+t2)+14utt(x2−ct2,−x2c+t2),∂∂xu0(x,t) =12ux(x2+ct2,x2c+t2)+12cut(x2+ct2,x2c+t2)   +12ux(x2−ct2,−x2c+t2)−12cut(x2−ct2,−x2c+t2),∂2∂x2u0(x,t) =14uxx(x2+ct2,x2c+t2)+12cuxt(x2+ct2,x2c+t2)  +14c2utt(x2+ct2,x2c+t2)+14uxx(x2−ct2,−x2c+t2)  −12cuxt(x2−ct2,−x2c+t2)+14c2utt(x2−ct2,−x2c+t2),

for all *x*, *t* ∈ ℝ. Hence, we know that

(19) $$\begin{array}{c} \frac{\partial^{2}}{\partial t^{2}}u_{0}\left(x,t\right)-c^{2}\frac{\partial^{2}}{\partial x^{2}}u_{0}\left(x,t\right)=0, \end{array}$$

for any *x*, *t* ∈ ℝ; that is, *u*_0_(*x*, *t*) is a solution of the wave equation (2).

**Corollary 2**

Given a constant *α* > 0, let a function *φ* : ℝ × ℝ → [0, *∞*) be given as

(20) $$\begin{array}{c} \varphi \left(x,t\right)=\alpha e^{-x^{2}-c^{2}t^{2}}. \end{array}$$

If a twice continuously differentiable function *u* : ℝ × ℝ → *ℂ* satisfies inequality (6), for all *x*, *t* ∈ ℝ, then there exists a solution *u*_0_ : ℝ × ℝ → *ℂ* of the wave equation (2) which satisfies

(21) $$\begin{array}{c} \left|u\left(x,t\right)-u_{0}\left(x,t\right)\right|\leq \frac{\alpha \pi }{8c^{2}}\left|\mathrm{erf}\left(\frac{x-ct}{\sqrt{2}}\right)\mathrm{erf}\left(\frac{x+ct}{\sqrt{2}}\right)\right|, \end{array}$$

for all *x*, *t* ∈ ℝ.

**Proof**

Since

(22) |∫0b∫0aφ(μ+ν2,μ−ν2c)dμdν| =|∫0b∫0aαe−μ2/2−ν2/2dμdν| =α|∫0be−ν2/2dν||∫0ae−μ2/2dμ| =2απ4|(2π∫0b/2e−ν2dν)(2π∫0a/2e−μ2dμ)| =απ2|erf⁡(b2)erf⁡(a2)|<∞,

for all *a*, *b* ∈ ℝ, in view of Theorem 1, we conclude that the statement of this corollary is true.
